# The Relationship of the Red Cell Distribution Width-to-Albumin Ratio and Other Inflammatory Markers With Cataracts: An Analysis of the NHANES Population

**DOI:** 10.1167/tvst.15.3.25

**Published:** 2026-03-25

**Authors:** Yao Kong, Zifeng Xu, Yanxin Xu, Shuoxian Chen, Yingying Liang, Xinrui Zou, Shitong Huang, Yu Jiang, Yunxia Leng, Zongyin Gao

**Affiliations:** 1 Department of Ophthalmology, Guangzhou First People's Hospital, Guangzhou Medical University, Guangzhou, China; 2 Department of Urology, The Third Affiliated Hospital, Sun Yat-sen University, Guangzhou, Guangdong, China; 3 Department of Ophthalmology, Guangzhou First People's Hospital, the Second Affiliated Hospital of South China University of Technology, Guangzhou, China; 4 Department of Ophthalmology, Guangzhou First People's Hospital, Guangdong Medical University, Guangzhou, China

**Keywords:** cataract, red cell distribution width, albumin, RAR, inflammation, biomarker, NHANES, oxidative stress, chronic inflammation

## Abstract

**Purpose:**

This study aimed to explore the relationship between the ratio of red cell distribution width to albumin (RAR) and cataract risk.

**Methods:**

We analyzed 13,031 participants from the National Health and Nutrition Examination Survey 1999–2008. The RAR was evaluated as a composite marker of systemic inflammation and nutritional status. Multivariable logistic regression and receiver operating characteristic analysis were used to assess the association and discriminative ability of the RAR compared with other inflammatory markers.

**Results:**

The findings indicated a positive link between higher RAR levels and the risk of cataracts, with a nonlinear relationship exhibiting an inverted U shape. Individuals in the higher quartiles of the RAR were observed to have a significantly greater risk of cataracts compared with those in the lower quartiles. The area under the curve for the RAR in predicting cataracts was determined to be 0.601, suggesting a greater predictive capability compared with other inflammatory markers, including the neutrophil-to-lymphocyte ratio and the Systemic Immune Inflammation Index.

**Conclusions:**

A higher RAR is significantly associated with moderate-to-severe cataracts. An RAR threshold of >3.025 may serve as a practical metric for identifying high-risk individuals, particularly among those aged ≥50 years.

**Translational Relevance:**

As a routine and cost-effective marker of inflammation and nutrition, the red cell distribution width-to-albumin ratio offers a practical tool to support cataract risk stratification and early identification in primary care settings.

## Introduction

Cataract is characterized by the clouding of the lens, which obstructs the normal passage of light and disrupts its proper focusing on the retina, ultimately leading to impaired vision.^1^ Cataracts are the leading cause of blindness worldwide, accounting for approximately 47.8% to 51.0% of all cases of blindness and approximately 80% of cases of reversible blindness.^2^^,^^3^ With the accelerating trend of global population aging, the incidence of cataracts is expected to rise annually. Although cataract surgery can significantly improve vision, high costs and a scarcity of qualified surgical professionals in certain regions continue to pose challenges to surgical accessibility. Moreover, patients may experience complications after cataract surgery, such as inflammation, dry eyes, macular edema, or posterior capsule opacification, all of which can greatly impact quality of life. Therefore, elucidating the underlying changes that lead to cataracts is crucial for developing effective preventive strategies.^4^

In addition to the well-recognized factor of population aging, oxidative stress, ultraviolet radiation, osmotic pressure, and other damaging factors, such as smoking and malnutrition, can also induce or accelerate lens opacification.^5^ Among these, malnutrition and inflammation play significant roles in the pathogenesis of cataracts.^6^ Inflammation exacerbates oxidative damage to the lens and epithelial cell apoptosis by activating signaling pathways and releasing reactive oxygen species and inflammatory factors, thereby promoting the occurrence and progression of cataracts.^7^ Therefore, inflammation, being a controllable and increasingly common risk factor, should be prioritized in initiatives aimed at averting the development and progression of cataracts.

In recent years, biomarkers obtained from whole blood parameters have surfaced as a valuable approach for the swift and convenient evaluation of systemic inflammation levels, thus improving research efficiency.^8^ Studies have demonstrated that several inflammatory markers in the serum of cataract patients—including interleukin-6 (IL-6), IL-1β, C-reactive protein, and tumor necrosis factor-α—are significantly higher than in healthy individuals.^9^ Several studies have examined the possible link between these inflammatory markers and cataracts. The Systemic Immune Inflammation Index (SII) and the neutrophil-lymphocyte ratio (NLR) have been identified as independent risk factors for cataracts in American adults.^10^^,^^11^ However, their restricted sensitivity and specificity, along with a primary emphasis on prognosis instead of predictive ability, limit their wider clinical use.

Red cell distribution width (RDW) and the RDW-to-albumin ratio (RAR) are novel inflammatory–nutritional composite biomarkers used to assess immune status and immune response, both of which are routine and widely available clinical parameters. The RDW reflects variability in red blood cell size, and its elevation may arise from erythropoietic disorders, changes in cell survival, or systemic inflammation. Additionally, an elevated RDW is linked to potential oxidative stress, inflammation, and resistance to microvascular blood flow.^12^^,^^13^ In the context of ophthalmic diseases, multiple studies have suggested that an elevated RDW is closely linked to various eye conditions, such as retinal vein occlusion, central retinal artery occlusion, and retinopathy of prematurity.^14^^–^^16^ Chen et al.^17^ found that an increased RDW is a risk factor for primary angle-closure glaucoma and correlates with the severity of the condition. Albumin, a key plasma protein, serves as an indicator of nutritional status and plays a role in regulating inflammatory responses. Research suggests that, as people age, the decrease in plasma albumin levels and the increase in free fatty acid concentrations are closely linked to the development of cataracts.^18^ Considering that inflammation, malnutrition, and aging are key factors linked to cataract development, integrating RDW and albumin into a single parameter—the RAR—may offer a more integrative measure of systemic inflammation and nutritional status.

Accordingly, this study aimed to explore the association between the RAR and cataract and to evaluate whether the RAR could serve as an easily obtainable marker reflecting systemic inflammatory and nutritional conditions relevant to cataract.

## Methods

### Study Population

The National Health and Nutrition Examination Survey (NHANES) is a continuous, publicly accessible national research initiative overseen by the Centers for Disease Control and Prevention (CDC), which offers researchers worldwide free access to its data. This project uses a sophisticated multistage probability sampling method to assess the nutritional status and overall health of both adults and children in the United States.

**Figure 1.**
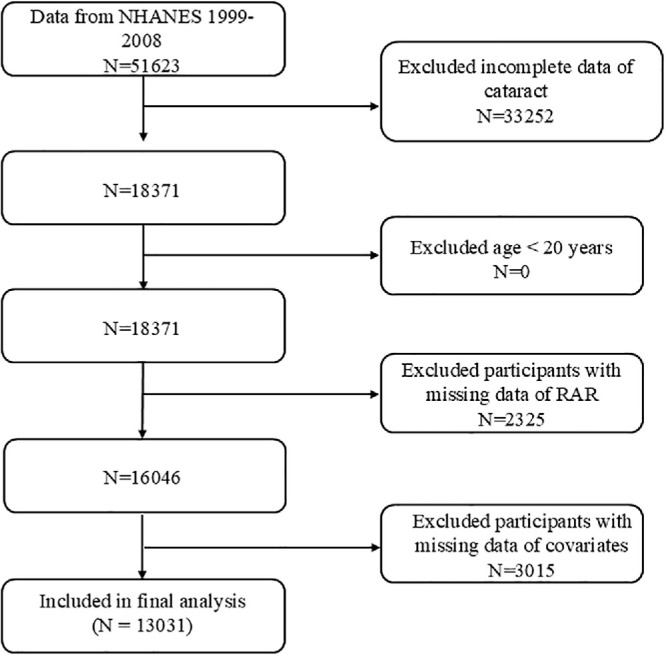
Flow chart of the study participants.

This research received ethical approval from the Research Ethics Review Board of the National Center for Health Statistics (https://www.cdc.gov/nchs/nhanes/irba98.htm). All participants provided written informed consent before data collection. The current analysis was based on NHANES survey waves conducted between 1999 and 2008, which included a total of 13,031 eligible participants (Fig. 1). This specific timeframe was selected because data on self-reported cataract surgery were consistently collected during this period, but were discontinued in subsequent cycles.

### Definition of Cataract and RAR

In NHANES, trained medical personnel used a Coulter analyzer to evaluate the RDW%, and serum albumin (g/dL) was measured using the DcX 800 two-color end point method (BCP complex) to calculate the RAR. The population was categorized into quartiles based on the RAR, with Q1 serving as the reference group.^19^^,^^20^ At the same time, a complete blood count was conducted using the Beckman Coulter DxH 800, providing measurements for monocytes (MC), lymphocytes (LC), neutrophils (NC), and platelets (PC). The MC-LC ratio (MLR) was derived from the ratio of MC to LC. SII was determined as PC × NC/LC, the systemic inflammation response index (SIRI) was computed as NC × MC/LC, the PC-LC ratio (PLR) was calculated as PC/LC, and the NLR was given by NC/LC.

Cataract status in this study was defined according to self-reported history of cataract surgery in NHANES. Participants were asked, “Have you ever had a cataract operation?” (VIQ070/VIQ071). Those who responded “yes” were classified as having a history of cataract, whereas those who responded “no” were classified as not having cataract. Because NHANES is a cross-sectional survey, it does not provide detailed clinical information such as preoperative lens status, onset, severity, or progression of cataract before surgery. Consequently, our definition mainly reflects surgically treated, clinically significant cataract, and likely under-represents undiagnosed or early-stage cataracts.^21^

### Selection of Covariates

The covariates considered in this study included demographic and health-related information, specifically: age, sex, race, education level, marital status, alcohol consumption status, smoking status, hyperlipidemia, hypertension, diabetes mellitus, body mass index (BMI), and the ratio of family income to the poverty line.

The poverty income ratio was classified into three groups: low income (<1.3), middle income (1.3–3.5), and high income (>3.5). Smoking status was categorized into three types: never smokers (lifetime smoking <100 cigarettes), former smokers (lifetime smoking of ≥100 cigarettes but not currently smoking), and current smokers (lifetime smoking of ≥100 cigarettes with daily use). Alcohol consumption was classified as yes or no, depending on whether the individual exceeded 12 standard drinks annually. The definitions of hypertension and hyperlipidemia are based on the article by Whelton et al.^22^

### Statistical Analyses

We adhered to the statistical analysis guidelines provided by the CDC and applied NHANES sampling weights to address the complex multistage cluster sampling data. Because our analysis combined five 2-year NHANES cycles (1999–2000, 2001–2002, 2003–2004, 2005–2006, and 2007–2008), we constructed a 10-year MEC examination weight by dividing the original 2-year MEC examination weight (WTMEC2YR) by five and used this derived weight (WTMEC10YR), together with SDMVPSU and SDMVSTRA, to specify the survey design. Categorical variables are reported as percentages, and continuous variables are described by the mean along with the standard error. Patients were categorized into four groups according to their RAR. Differences among the different RAR quartile groups were analyzed using weighted χ^2^ tests for categorical variables and weighted *t* tests for continuous data.

Weighted multivariable logistic regression models were used to assess the independent association of RAR quartiles with cataracts, presenting the results as odds ratios (ORs) along with their respective 95% confidence intervals (CIs). Sensitivity analyses were performed by repeating the fully adjusted survey-weighted models in participants aged ≥50 years. To analyze nonlinear relationships, we used survey-weighted generalized additive models and applied smooth curve fitting. The log-likelihood ratio test was used to compare the fitting differences between piecewise regression models and linear models, exploring potential threshold effects.

Receiver operating characteristic (ROC) curves were used to assess the predictive capability of age, the RAR, and other inflammatory indicators in both single‑predictor and multivariable models. For the evaluation of multivariable logistic regression models, ROC analyses were performed using predicted probabilities derived from the survey-weighted models (svyglm) to ensure population representativeness. Additionally, standard unweighted ROC analyses were used to determine the optimal clinical thresholds for the RAR and other biomarkers at the individual level. The DeLong test was performed to assess the statistical significance of the differences in area under the curves (AUCs) between models. All statistical tests were two-sided, and a *P* value of <0.05 was considered statistically significant.

## Results

### Participant Characteristics

The study population comprised 13,031 participants, including 6469 men (48.0%) and 6562 women (52.0%), with a mean age of 55.95 ± 17.57 years. The mean RAR level for the overall cohort was 3.09 ± 0.45. Detailed baseline characteristics are summarized in Table 1.

**Table 1.** Baseline Characteristics According to RAR

	Quartiles of RAR					
Characteristics	Overall	Q1 (<2.95)	Q2 (2.95–3.16)	Q3 (3.16–3.40)	Q4 (>3.40)	*P* Value
No.	13,031	5515	3269	2046	2201	
NLR	2.24 ± 1.15	2.14 ± 1.03	2.21 ± 1.06	2.26 ± 1.15	2.65 ± 1.63	<0.001
MLR	0.29 ± 0.12	0.28 ± 0.11	0.29 ± 0.12	0.29 ± 0.13	0.32 ± 0.16	<0.001
PLR	140.49 ± 55.96	136.75 ± 49.91	137.94 ± 52.09	142.12 ± 58.37	159.03 ± 76.57	<0.001
SIRI	1.27 ± 0.86	1.20 ± 0.75	1.26 ± 0.81	1.29 ± 0.88	1.55 ± 1.21	<0.001
SII	602.73 ± 366.02	567.38 ± 308.02	589.68 ± 320.14	616.32 ± 351.53	757.82 ± 582.51	<0.001
Age, years						<0.001
20–44	3437 (31.0)	1760 (36.5)	699 (25.9)	396 (24.1)	582 (26.4)	
41–60	4975 (44.4)	2228 (45.5)	1293 (45.9)	737 (43.2)	717 (38.8)	
>60	4619 (24.6)	1527 (18.0)	1277 (28.2)	913 (32.7)	902 (34.8)	
Gender						<0.001
Male	6469 (48.0)	3288 (58.2)	1581 (44.5)	835 (33.5)	765 (30.5)	
Female	6562 (52.0)	2227 (41.8)	1688 (55.5)	1211 (66.5)	1436 (69.5)	
Race						<0.001
Mexican American	2473 (6.45)	1168 (6.87)	612 (6.22)	331 (5.29)	362 (6.50)	
Other Hispanic	746 (4.11)	300 (3.87)	212 (4.31)	115 (4.13)	119 (4.68)	
Non-Hispanic White	6992 (75.31)	3254 (79.15)	1800 (76.29)	1036 (72.53)	902 (60.83)	
Non-Hispanic Black	2374 (9.36)	573 (4.80)	541 (8.78)	501 (14.00)	759 (23.81)	
Other Race, Including Multiracial	446 (4.78)	220 (5.31)	104 (4.41)	63 (4.05)	59 (4.18)	
Education						<0.001
Less than high school	4066 (19.7)	1540 (16.7)	1045 (21.1)	674 (21.7)	807 (27.0)	
High school or GED	3108 (25.4)	1309 (24.6)	768 (25.2)	523 (29.8)	508 (24.3)	
Above high school	5857 (54.9)	2666 (58.7)	1456 (53.8)	849 (48.4)	886 (48.7)	
Marital status						<0.0001
Married or living with partner	8158 (66.3)	3700 (69.9)	2079 (67.0)	1176 (61.3)	1203 (56.1)	
Unmarried or other	4873 (33.7)	1815 (30.1)	1190 (33.0)	870 (38.7)	998 (43.9)	
PIR						<0.0001
Low income	3536 (17.9)	1295 (14.7)	909 (18.7)	566 (19.1)	766 (27.6)	
Medium income	5100 (36.2)	2115 (35.2)	1248 (35.0)	874 (40.1)	863 (38.2)	
High income	4395 (46.0)	2105 (50.1)	1112 (46.3)	606 (40.8)	572 (34.2)	
BMI (kg/m^2^)						<0.0001
Normal/underweight	3700 (30.8)	1891 (36.9)	868 (27.3)	468 (23.7)	473 (21.5)	
Overweight	4804 (35.5)	2240 (38.9)	1204 (34.6)	707 (32.0)	653 (27.8)	
Obese	4527 (33.6)	1384 (24.2)	1197 (38.1)	871 (44.3)	1075 (50.6)	
Smoking status						0.529
Never smoker	6423 (48.9)	2707 (48.9)	1605 (48.6)	997 (49.5)	1114 (49.1)	
Former smoker	4066 (30.0)	1737 (30.5)	1017 (29.4)	628 (28.3)	684 (30.9)	
Current smoker	2542 (21.1)	1071 (20.6)	647 (22.0)	421 (22.2)	403 (19.9)	
Drinking status						<0.001
Yes	8894 (72.2)	4114 (78.0)	2188 (69.9)	1291 (65.2)	1301 (61.5)	
No	4137 (27.8)	1401 (22.0)	1081 (30.1)	755 (34.8)	900 (38.5)	
Hypertension						<0.001
Yes	7802 (54.7)	3034 (50.2)	2056 (58.2)	1325 (59.2)	1387 (61.5)	
No	5229 (45.3)	2481 (49.8)	1213 (41.8)	721 (40.8)	814 (38.5)	
Hyperlipidemia						0.131
Yes	9085 (69.7)	3837 (69.0)	2312 (70.9)	1432 (71.3)	1504 (68.1)	
No	3946 (30.3)	1678 (31.0)	957 (29.1)	614 (28.7)	697 (31.9)	
Diabetes mellitus						<0.001
Yes	2217 (12.7)	696 (8.7)	555 (14.0)	399 (16.3)	567 (22.4)	
No	10814 (87.3)	4819 (91.3)	2714 (86.0)	1647 (83.7)	1634 (77.6)	
Cataract						<0.001
Yes	1624 (9.0)	474 (5.9)	439 (10.0)	335 (12.7)	376 (15.5)	
No	11407 (91.0)	5041 (94.1)	2830 (90.0)	1711 (87.3)	1825 (84.5)	

PIR, poverty income ratio.

Values are mean ± standard deviation or number (%). All estimates were calculated using complex survey designs, with analysis of variance or χ² tests applied as appropriate.

**Table 2.** ORs and 95% CIs for Cataract by RAR Quartiles

		Model 1		Model 2		Model 3	
Index	Continuous or Categories	OR (95% CI)	*P* Value	OR (95% CI)	*P* Value	OR (95% CI)	*P* Value
RAR	Continuous variable	2.07 (1.82–2.36)	<0.001	1.38 (1.17–1.63)	<0.001	1.33 (1.13–1.58)	<0.001
	Q1	Reference		Reference		Reference	
	Q2	1.81 (1.50–2.20)	<0.001	1.14 (0.91–1.41)	0.247	1.13 (0.91–1.40)	0.277
	Q3	2.34 (1.92–2.84)	<0.001	1.24 (0.96–1.60)	0.102	1.23 (0.95–1.59)	0.120
	Q4	2.99 (2.47–3.60)	<0.001	1.56 (1.24–1.97)	<0.001	1.48 (1.17–1.86)	0.001
	*P* for trend	<0.001		<0.001		0.003	

Model 1 was unadjusted.

Model 2 was adjusted for age, race, education, marital status, poverty income ratio, gender, and BMI.

Model 3 was further adjusted for smoking status, alcohol consumption, hypertension, hyperlipidemia, and diabetes.

When participants were stratified by RAR quartiles, the prevalence of cataract in the highest quartile (Q4) was 15.5%, significantly exceeding the 9.0% observed in the lowest quartile (Q1) (*P* < 0.001). Demographic analysis revealed distinct patterns across quartiles: participants with higher RAR levels tended to be older, were more likely to identify as other Hispanic race, and had greater proportions of unmarried individuals. Furthermore, an elevated RAR was associated with lower educational attainment, a progressively increasing prevalence of low income, and a higher BMI. Regarding lifestyle and clinical factors, higher RAR quartiles correlated with increased rates of alcohol consumption, hypertension, and diabetes.

### Relationship Between RAR and Cataract

By applying a weighted multivariable logistic regression approach, we aimed to evaluate the independent relationship between quartiles of RAR and the risk of developing cataracts. In both the unadjusted model (model 1) and the model adjusted for demographic factors (model 2), the RAR was positively associated with cataract risk, showing an increasing risk with higher RAR quartiles (Table 2). Furthermore, after incorporating lifestyle factors and comorbid conditions in model 3, the RAR maintained a significant association with cataract risk as a continuous variable (OR, 1.33; 95% CI, 1.13–1.58; *P* < 0.001).

In the quartile analysis, the risk of cataracts in the Q4 was significantly greater compared with the Q1 (OR, 1.48; 95% CI, 1.17–1.86; *P* = 0.001). Additionally, trend tests showed that the dose–response relationship was statistically significant (*P* for trend = 0.003).

A sensitivity analysis restricted to participants aged ≥50 years—the population most susceptible to cataracts—confirmed these findings. In the fully adjusted model for this subgroup, each 1-unit increase in RAR was associated with 34% higher odds of cataract (OR, 1.34; 95% CI, 1.13–1.60; *P* < 0.01) (Supplementary Table S1). In an additional model excluding potential mediators (BMI, hypertension, diabetes, and hyperlipidemia), the association was slightly stronger (OR, 1.38; 95% CI, 1.17–1.62), suggesting that overadjustment may partially attenuate the observed effect. These consistent findings across different adjustment strategies support a robust, independent relationship between a higher RAR and an increased cataract risk.

**Figure 2.**
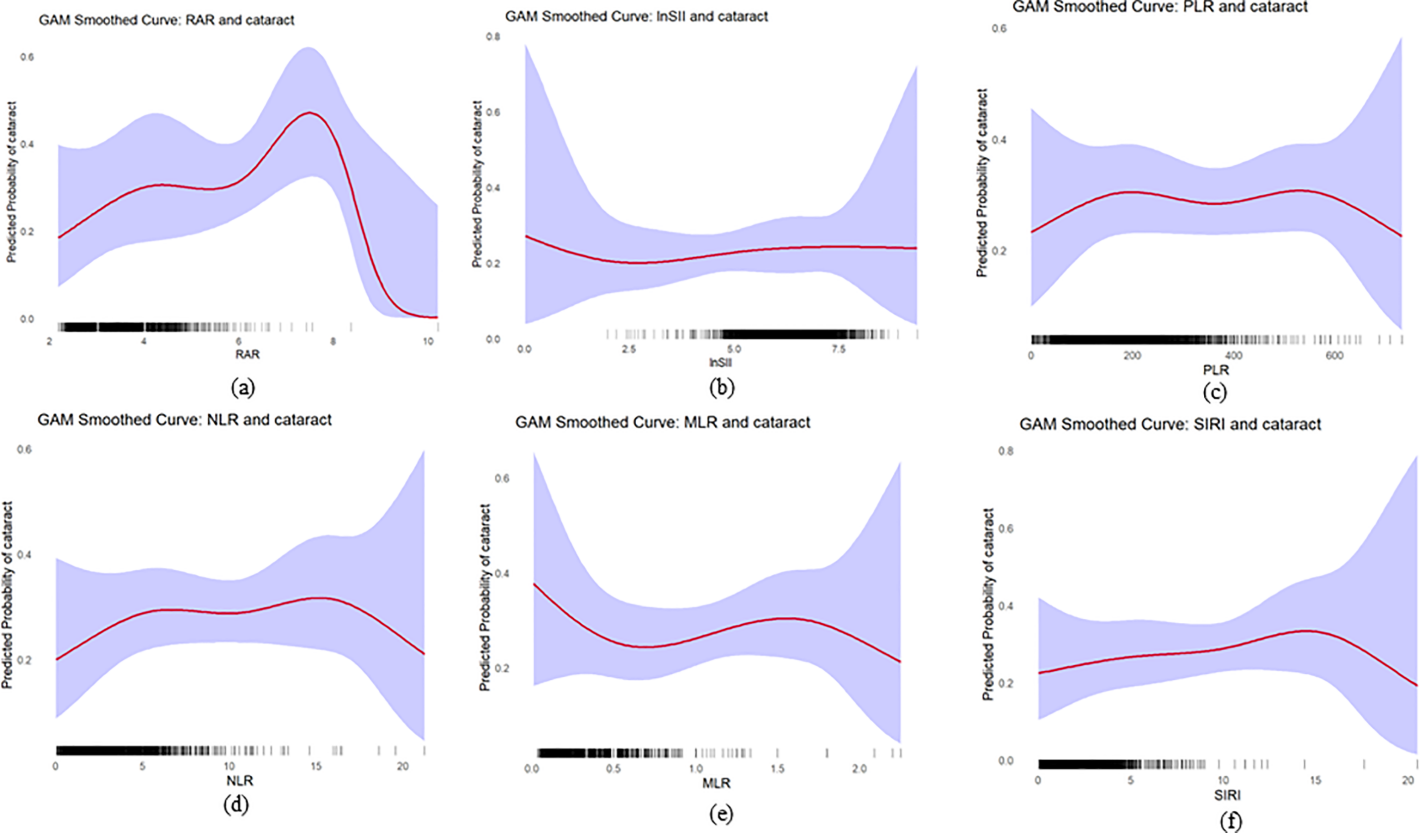
Analysis of RAR and five other markers related to cataracts using generalized additive model smooth curve fitting. The rug plot on the *x* axis (each *black tick* represents one participant) illustrates the data distribution across RAR values. And the shaded purple area denotes the 95% CI. (**a**) RAR and cataracts. (**b**) lnSII and cataracts. (**c**) PLR and cataracts. (d) NLR and cataracts. (**e**) MLR and cataracts. (**f**) SIRI and cataracts.

Additionally, using generalized additive models and smooth curve analysis (Fig. 2), we discovered a nonlinear relationship between RAR and cataract risk, which exhibited an inverted U-shaped pattern. At lower RAR levels, the risk of cataracts gradually increased, peaking before decreasing. The ln-SII showed a monotonic decrease; the PLR and SIRI curves were nearly flat. In contrast, the NLR and MLR exhibited a U shape or turning point; however, improvements in the models at these turning points were not statistically significant. Piecewise regression further confirmed the nonlinear relationships between the other indicators and cataracts, and only the RAR demonstrated a significant statistical meaning (Table 3).

**Table 3.** Threshold Effect Analysis of RAR and Other Biomarkers On Cataract Risk

	RAR	lnSII	PLR	NLR	MLR	SIRI
Standard linear model						
OR (95% CI)	1.36 (1.15–1.60)	1.09 (0.94–1.26)	1.00 (0.999–1.001)	1.00 (0.96–1.07)	1.08 (0.66–1.76)	1.04 (0.96–1.12)
*P* value	<0.001	0.261	0.737	0.677	0.756	0.336
Fitting by two-piecewise linear model						
Breakpoint (K)	4.6	3.0	137.2	15.0	0.9	15.0
OR1 (<K)	1.56 (1.22 –2)	0.07 (0.01–0.79)	1.00 (0.996–1.002)	1.02 (0.96–1.09)	1.22 (0.69–2.14)	1.06 (0.98–1.14)
OR2 (>K)	0.62 (0.31 –1.21)	1.11 (0.96–1.29)	1.001(0.999–1.002)	0.51 (0.24–1.1)	0.47 (0.07–3.09)	0.01 (0–0.03)
OR2/OR1	0.39	15.18	1	0.50	0.39	0.01
Logarithmic likelihood ratio test *P* value	0.017	0.11	0.737	0.174	0.376	0.11

Notably, The apparent decrease in cataract risk at the extreme upper tail of the RAR range appears to be driven by severe data sparsity (only a few participants had RAR values of >7.0), resulting in wide CIs and statistical instability in this region.

### Subgroup Analysis

**Figure 3.**
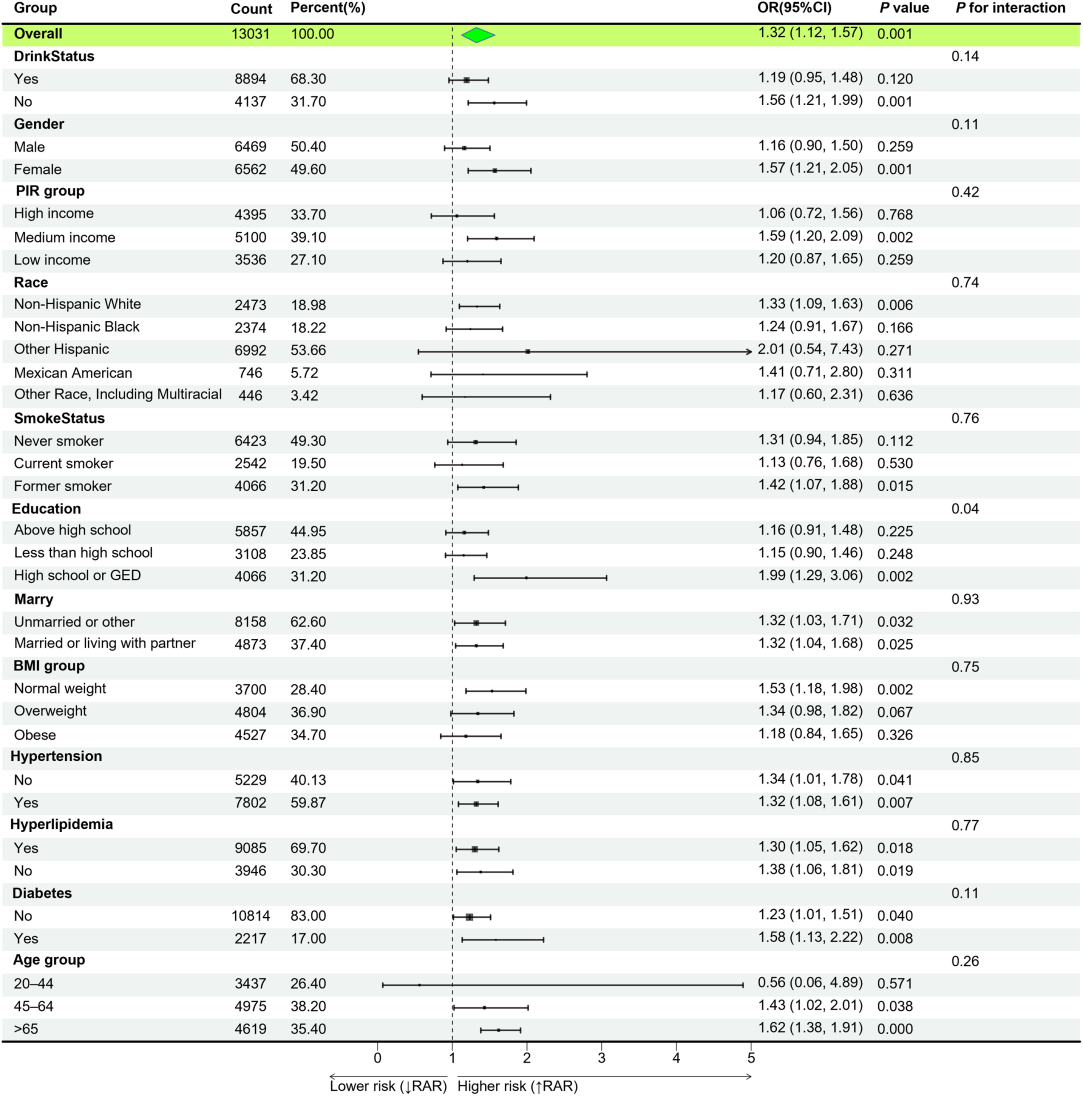
Subgroup analysis of the associations between RAR and cataract. PIR, poverty income ratio.

To assess the stability of the relationship between the RAR and cataracts across different populations, we conducted interaction tests within subgroups classified by factors such as alcohol consumption, sex, income level, education, race/ethnicity, smoking habits, and marital status (Fig. 3). Before correction, the interaction *P* value was 0.027 for the education subgroup, whereas the *P* values for all other subgroups were >0.05. After Bonferroni correction, the relationship between the RAR and cataract risk remained stable across all subgroups, with no significant interactions found (all interaction *P* values > 0.05).

### ROC Curve Analysis

**Figure 4.**
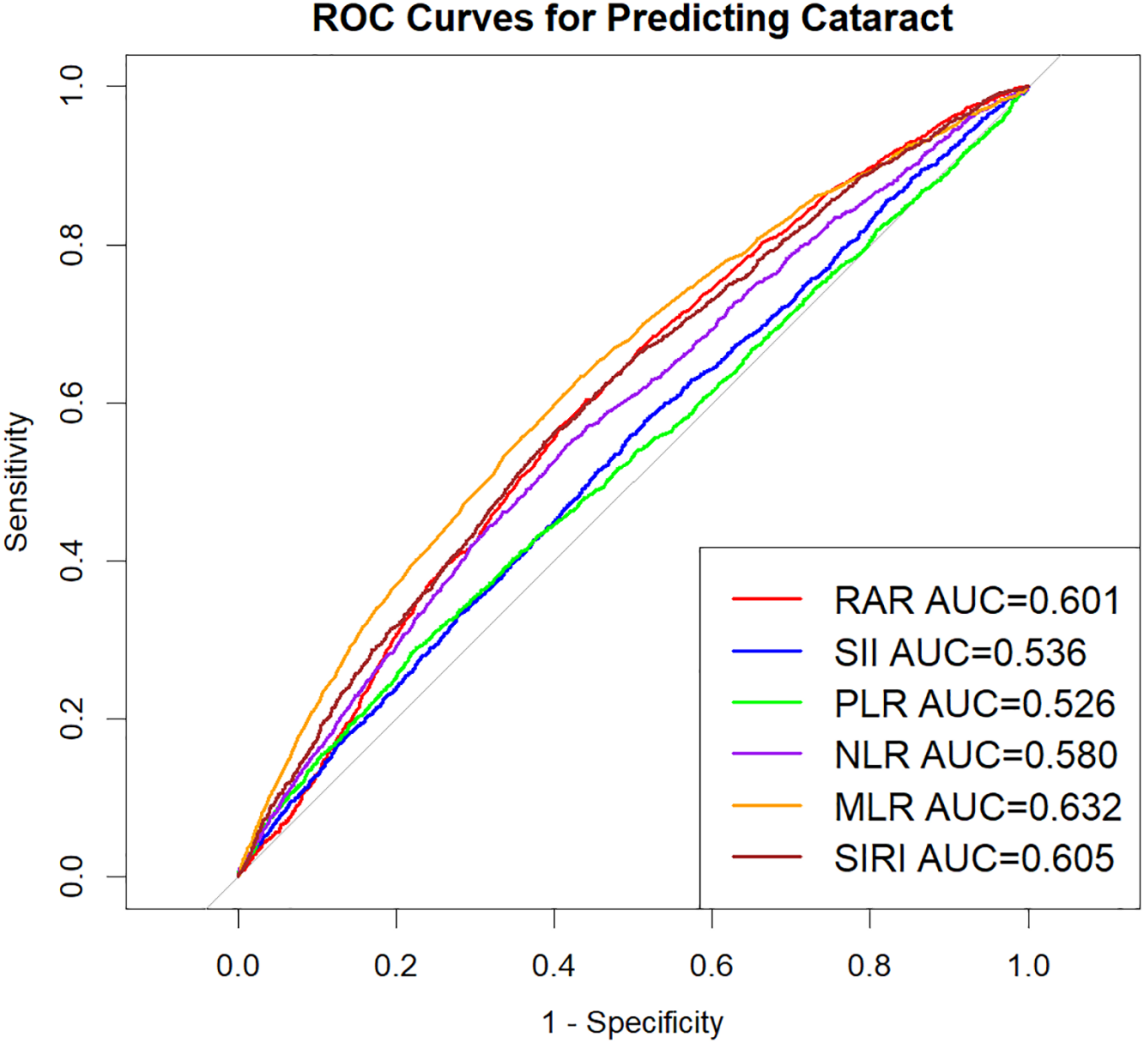
The role of six markers in cataract diagnosis as illustrated by ROC curves.

Figure 4 compares the ROC curves and AUC values of RAR against other inflammatory biomarkers (SII, PLR, NLR, MLR, and SIRI) in predicting cataracts.

Among the inflammatory indices, the RAR (AUC = 0.601) showed a discriminative ability statistically comparable with the SIRI (AUC = 0.605; *P* = 0.725) and superior to the NLR, SII, and PLR (all *P* < 0.05) (Table 4), although it ranked below the MLR (AUC = 0.632). Furthermore, regarding its incremental value, adding the RAR to established demographic risk factors yielded a statistically significant improvement in discrimination (DeLong's test: Z = −2.67; *P* = 0.007) (Supplementary Fig. S1), even though the absolute increase in the AUC was modest due to the dominant predictive contribution of age. Taken together, these findings indicate that the RAR possesses meaningful incremental predictive potential.

**Table 4.** Comparison of AUC Values for RAR and Other Inflammatory Markers

Variables	AUC	95% CI Low	95% CI Up	Best Threshold	Specificity	Sensitivity	*P* Value for Difference
RAR	0.601	0.587	0.615	3.025	0.559	0.603	Reference
SII	0.536	0.521	0.551	511.676	0.504	0.558	<0.001
PLR	0.526	0.510	0.542	167.633	0.774	0.291	<0.001
NLR	0.580	0.565	0.595	2.153	0.582	0.547	0.029
MLR	0.632	0.618	0.647	0.281	0.589	0.611	0.002
SIRI	0.605	0.590	0.619	1.184	0.598	0.565	0.725

## Discussion

This study, based on data from NHANES collected between 1999 and 2008, investigates the relationship between RAR and the risk of cataracts. The findings indicate that the RAR is positively correlated with cataract risk, with the likelihood of developing cataracts gradually increasing across higher RAR quartiles. After adjusting for covariates, the RAR remained significantly associated with cataract risk as a continuous variable. In terms of predictive performance, although the RAR alone demonstrated modest discriminative ability (AUC = 0.601), it maintained a significant association in the fully adjusted model (AUC = 0.883), suggesting that the RAR contributes independent information beyond demographic and metabolic factors. These findings suggest that an elevated RAR may reflect systemic inflammatory and nutritional disturbances that commonly coexist with cataract.

Lim et al^23^ proposed that chronic inflammation can promote oxidative stress, which in turn may alter lens proteins and contribute to lens opacification. In addition, immune responses involving cytokines and other inflammatory mediators have been reported to be closely related to cataract occurrence and progression.^24^ Peripheral blood–derived inflammatory indices, initially developed to assess systemic inflammation and stress in critically ill patients, have since been applied to a wide range of acute and chronic diseases because they are easily obtainable, reproducible, and inexpensive.^25^ Several count-based inflammatory markers have been investigated as tools for risk stratification, prognosis, and treatment response monitoring in different clinical settings.^26^ Ratios such as NLR and PLR are widely used to evaluate systemic inflammatory burden and to identify patients at higher inflammatory risk.^10^ MLR reflects circulating immune status,^27^ and SII, which integrates PC, NC, and LC counts, provides a more comprehensive measure of immune-inflammatory activation. In an analysis of NHANES data from 2005–2008, Li et al. reported that NLR, PLR, and SII were each associated with the presence of cataract, with AUC values of 0.549 for SII and 0.603 for NLR, findings that are broadly consistent with our results. RAR, as a newer composite marker integrating inflammatory and nutritional information, has also gained attention in ophthalmic research using NHANES data. Zhao et al. found that RAR was independently associated with diabetic retinopathy,^28^ and An et al. described a positive linear association between RAR and age-related macular degeneration, suggesting that RAR might serve as a quantitative indicator of retinal inflammatory damage.^29^ In the present study involving 13,031 NHANES participants, RAR showed a predominantly positive association with cataract risk. It demonstrated discriminative ability comparable to or superior to other inflammatory markers and, importantly, offered statistically significant incremental predictive value beyond demographics. However, the inverted “U” pattern should be interpreted with caution; the main robust finding is a predominantly positive association between RAR and cataract risk across most of the observed range. The apparent decline in risk at very high RAR levels may hint at compensatory mechanisms or a possible “survivor bias” under extreme pathophysiological conditions, but this is speculative. Future studies with larger samples, especially including more individuals with very high RAR levels, are needed to confirm these observations and better define the dose–response relationship between RAR and cataracts.

The gradual clouding of the lens in cataracts is primarily driven by the accumulation of oxidative stress and protein aggregation within the lens fibers, both of which are strongly linked to inflammation.^1^^,^^7^ Reactive oxygen species (ROS), major byproducts generated during phototransduction and metabolic processes, can activate the expression of pro-inflammatory cytokines, promote immune responses, and create a destructive cycle of inflammation and oxidative damage, thereby facilitating cataract formation.^30^^,^^31^ Chen et al. found that patients with cataracts exhibited elevated concentrations of IL-1, IL-1β, IL-6, and tumor necrosis factor-α in their vitreous fluid, reinforcing the notion that inflammation plays a significant role in the development of cataracts.^32^ Red blood cell production, lifespan, and deformability can be influenced by various factors, including inflammatory triggers, infections, and nutritional or metabolic issues, resulting in elevated RDW levels.^12^^,^^33^ At the same time, inflammatory responses can increase capillary permeability, leading to albumin leakage and ultimately resulting in lower serum albumin levels.^33^^,^^34^ As a key biomarker, serum albumin plays a vital role in evaluating both nutritional health and inflammatory conditions. As age increases, a decline in serum albumin concentration may elevate intraocular free fatty acid concentrations. This alteration can compromise the vascular-albumin barrier in the eye, potentially leading to mitochondrial dysfunction and the death of lens epithelial cells, which ultimately contributes to the development of cataracts.^18^ As a novel biomarker, RAR provides valuable insights for clinicians regarding the early prevention and intervention of cataracts. In our study, we found that the Q4 group significantly increased the risk of cataracts compared with the Q1. One possible explanation for this finding is that elevated RAR levels, influenced by increased RDW and/or hypoalbuminemia, indicate a state of chronic inflammation and malnutrition. This condition may exacerbate oxidative stress and immune dysregulation—crucial mechanisms in the development of cataracts. However, since our study did not directly measure lens protein oxidation or intraocular inflammatory levels, these mechanistic links remain speculative and represent hypotheses for future investigation.

This study has several limitations. Firstly, we encountered challenges in accessing essential data on confounding factors related to cataracts, such as light exposure, genetic predispositions, and levels of circulating steroid hormones, as this information is not included in the NHANES database. Secondly, the study's cross-sectional design limits our capacity to establish causal relationships and leaves open the possibility of reverse causation. Additionally, our identification of cataracts relied on whether participants had undergone cataract surgery, which limits the comprehensiveness of cataract screening. As a result, some individuals with undiagnosed or early-stage cataracts might have unintentionally been categorized into the control group. Furthermore, the lack of details regarding the extent of lens opacification hindered our evaluation of cataract severity, thus restricting our ability to provide a nuanced assessment of the diagnostic significance of risk factors for cataracts.

Giving these limitations, our findings should be interpreted as associative rather than causal. Prospective longitudinal studies with more detailed phenotyping and comprehensive assessment of potential confounders are needed to confirm these associations and clarify their temporal relationships. Furthermore, we conducted a sensitivity analysis restricted to older adults (aged ≥50 years). This analysis yielded findings consistent with our primary results, suggesting that potential misclassification due to the surgery-based definition did not substantially alter the observed associations (Supplementary Table S1)

Despite the aforementioned limitations, RAR, as a novel biomarker, may provide a valuable tool for the early identification and timely intervention of high‑risk cataract patients, thereby enhancing clinical prevention strategies and potentially reducing the incidence of cataracts. Specifically, an RAR value greater than 3.025 may serve as a practical alert threshold for risk stratification, particularly among individuals aged ≥50 years.

## Conclusions

The results of this study indicate that the ratio of RAR is positively associated with the presence of moderate-to-severe cataract, with the likelihood of having cataracts being higher at elevated RAR levels. More longitudinal studies are needed to confirm this relationship and clarify its temporal sequence. As a novel biomarker, RAR offers a valuable tool for the early identification and intervention of high-risk cataract patients. This could enhance clinical prevention strategies and ultimately reduce the incidence of cataracts.

## Supplementary Material

Supplement 1

## References

[1] Liu YC, Wilkins M, Kim T, Malyugin B, Mehta JS. Cataracts. *Lancet*. 2017; 390;600–612. doi:10.1016/s0140-6736(17)30544-5. 28242111

[2] Khairallah M, Kahloun R, Bourne R, et al. Number of people blind or visually impaired by cataract worldwide and in world regions, 1990 to 2010. *Invest Ophthalmol Vis Sci*. 2015; 56;6762–6769. doi:10.1167/iovs.15-17201. 26567788

[3] Flaxman SR, Bourne RRA, Resnikoff S, et al. Global causes of blindness and distance vision impairment 1990-2020: a systematic review and meta-analysis. *Lancet Global Health*. 2017; 5;e1221–e1234. doi:10.1016/s2214-109x(17)30393-5. 29032195

[4] Marques AP, Ramke J, Cairns J, et al. The economics of vision impairment and its leading causes: a systematic review. *EClinicalMedicine*. 2022; 46;101354. doi:10.1016/j.eclinm.2022.101354. 35340626 PMC8943414

[5] West SK, Valmadrid CT. Epidemiology of risk factors for age-related cataract. *Surv Ophthalmol*. 1995; 39;323–334. doi:10.1016/s0039-6257(05)80110-9. 7725232

[6] Böhm EW, Buonfiglio F, Voigt AM, et al. Oxidative stress in the eye and its role in the pathophysiology of ocular diseases. *Redox biology*. 2023; 68;102967. doi:10.1016/j.redox.2023.102967. 38006824 PMC10701459

[7] Medoro A, Davinelli S, Scuderi L, Scuderi G, Scapagnini G, Fragiotta S. Targeting senescence, oxidative stress, and inflammation: quercetin-based strategies for ocular diseases in older adults. *Clin Interv Aging*. 2025; 20;791–813. doi:10.2147/cia.S516946. 40503074 PMC12155388

[8] Tukenmez Dikmen N, Un Y. Systemic immuno-inflammatory index in patients with pseudoexfoliation syndrome and pseudoexfoliative glaucoma. *Ther Adv Ophthalmol*. 2023; 15;25158414231197072. doi:10.1177/25158414231197072. 37720205 PMC10504835

[9] Dong Y, Mu GY, Chen F, Zhao RL, Wang M, Wang B. Correlation between MMP-2 gene polymorphism and cataract susceptibility. *Eur Rev Med Pharmacol Sci*. 2019; 23;3167–3172. doi:10.26355/eurrev_201904_17674. 31081067

[10] Li B, Hou X, Ning B, et al. Predictive role of the peripheral blood inflammation indices neutrophil-to-lymphocyte ratio (NLR), platelet-to-lymphocyte ratio (PLR), and systemic immunoinflammatory index (SII) for age-related cataract risk. *PLoS One*. 2024; 19;e0313503. doi:10.1371/journal.pone.0313503. 39556543 PMC11573120

[11] Huang J, Wu H, Yu F, et al. Association between systemic immune-inflammation index and cataract among outpatient US adults. *Front Med*. 2024; 11;1469200. doi:10.3389/fmed.2024.1469200. PMC11445128 39359932

[12] Salvagno GL, Sanchis-Gomar F, Picanza A, Lippi G. Red blood cell distribution width: a simple parameter with multiple clinical applications. *Crit Rev Clin Lab Sci*. 2015; 52;86–105. doi:10.3109/10408363.2014.992064. 25535770

[13] Akpinar I, Sayin MR, Gursoy YC, et al. Plateletcrit and red cell distribution width are independent predictors of the slow coronary flow phenomenon. *J Cardiol*. 2014; 63;112–118. doi:10.1016/j.jjcc.2013.07.010. 24012331

[14] Elbeyli A, Kurtul BE, Ozcan DO, Ozcan SC, Dogan E. assessment of red cell distribution width, platelet/lymphocyte ratio, systemic immune-inflammation index, and neutrophil/lymphocyte ratio values in patients with central retinal artery occlusion. *Ocul Immunol Inflamm*. 2022; 30;1940–1944. doi:10.1080/09273948.2021.1976219. 34524949

[15] Çömez A, Yurttutan S, Seringec Akkececi N, et al. Red cell distribution width and its association with retinopathy of prematurity. *Int Ophthalmol*. 2021; 41;699–706. doi:10.1007/s10792-020-01627-7. 33118094

[16] Ozkok A, Nesmith BLW, Schaal S. Association of red cell distribution width values with vision potential in retinal vein occlusion. *Ophthalmol Retina*. 2018; 2;582–586. doi:10.1016/j.oret.2017.09.018. 31047612

[17] Chen Q, Zhao B, Wang MY, et al. Associations between the red blood cell distribution width and primary angle-closure glaucoma: a potential for disease prediction. *EPMA J*. 2019; 10;185–193. doi:10.1007/s13167-019-00166-1. 31258822 PMC6562012

[18] Glaesser D, Iwig M. Increased molar ratio of free fatty acids to albumin in blood as cause and early biomarker for the development of cataracts and Alzheimer's disease. *Exp Eye Res*. 2024; 243;109888. doi:10.1016/j.exer.2024.109888. 38583754

[19] Yu B, Li M, Yu Z, et al. Red blood cell distribution width to albumin ratio (RAR) is associated with low cognitive performance in American older adults: NHANES 2011–2014. *BMC Geriatr*. 2025; 25;157. doi:10.1186/s12877-025-05800-4. 40055657 PMC11887108

[20] Zhu XF, Hu YQ, Long ZW, Cao MZ. Association between RAR and the prevalence and prognosis of depression: a population-based study. *J Affect Disord*. 2025; 380;1–9. doi:10.1016/j.jad.2025.03.100. 40120147

[21] Li N, Fan Y, Li J, Guo J, Wang J, Gao Z. Cross-sectional association of oxidative balance score with cataract among US adults: NHANES 1999–2008. *Front Nutr*. 2025; 12;1555631. doi:10.3389/fnut.2025.1555631. 40201590 PMC11975577

[22] Whelton PK, Carey RM, Aronow WS, et al. 2017 ACC/AHA/AAPA/ABC/ACPM/AGS/APhA/ASH/ASPC/NMA/PCNA guideline for the prevention, detection, evaluation, and management of high blood pressure in adults: a report of the American College of Cardiology/American Heart Association Task Force on Clinical Practice Guidelines. *Circulation*. 2018; 138;e484–e594. doi:10.1161/cir.0000000000000596. 30354654

[23] Lim JC, Caballero Arredondo M, Braakhuis AJ, Donaldson PJ. Vitamin C and the lens: new insights into delaying the onset of cataract. *Nutrients*. 2020; 12;3142. doi:10.3390/nu12103142. 33066702 PMC7602486

[24] Li X, Du GL, Wu SN, et al. Association between Systemic Immune Inflammation Index and cataract incidence from 2005 to 2008. *Sci Rep*. 2025; 15;499. doi:10.1038/s41598-024-84204-7. 39747967 PMC11696561

[25] Bhikram T, Sandor P. Neutrophil-lymphocyte ratios as inflammatory biomarkers in psychiatric patients. *Brain Behav Immunity*. 2022; 105;237–246. doi:10.1016/j.bbi.2022.07.006. 35839998

[26] Ravindranathan D, Master VA, Bilen MA. Inflammatory markers in cancer immunotherapy. *Biology*. 2021; 10;325. doi:10.3390/biology10040325. 33924623 PMC8069970

[27] Ji H, Niu X, Yin L, et al. Ratio of immune response to tumor burden predicts survival via regulating functions of lymphocytes and monocytes in diffuse large B-cell lymphoma. *Cell Physiol Biochem*. 2018; 45;951–961. doi:10.1159/000487288. 29428948

[28] Zhao F, Liu M, Kong L. Association between red blood cell distribution width-to-albumin ratio and diabetic retinopathy. *J Clin Lab Anal*. 2022; 36;e24351. doi:10.1002/jcla.24351. 35285094 PMC8993659

[29] An N, Zeng B, Liu Z, et al. Red blood cell distribution width-to-albumin ratio and its association with age-related macular degeneration: a population-based cross-sectional study. *Front Med*. 2025; 12;1510756. doi:10.3389/fmed.2025.1510756. PMC12037637 40303372

[30] Morgan MJ, Liu ZG. Crosstalk of reactive oxygen species and NF-κB signaling. *Cell Res*. 2011; 21;103–115. doi:10.1038/cr.2010.178. 21187859 PMC3193400

[31] Xiao R, Huang X, Gao S, et al. Microglia in retinal diseases: From pathogenesis towards therapeutic strategies. *Biochem Pharmacol*. 2024; 230;116550. doi:10.1016/j.bcp.2024.116550. 39307318

[32] Chen W, Lin H, Zhong X, et al. Discrepant expression of cytokines in inflammation- and age-related cataract patients. *PLoS One*. 2014; 9;e109647. doi:10.1371/journal.pone.0109647. 25303043 PMC4193817

[33] Soeters PB, Wolfe RR, Shenkin A. Hypoalbuminemia: pathogenesis and clinical significance. *JPEN J Parenter Enter Nutr*. 2019; 43;181–193. doi:10.1002/jpen.1451. PMC7379941 30288759

[34] Sheinenzon A, Shehadeh M, Michelis R, Shaoul E, Ronen O. Serum albumin levels and inflammation. *Int J Biol Macromol*. 2021; 184;857–862. doi:10.1016/j.ijbiomac.2021.06.140. 34181998

