# Supplementary material for: The Relationship of the Red Cell Distribution Width-to-Albumin Ratio and Other Inflammatory Markers With Cataracts: An Analysis of the NHANES Population
Source: Transl Vis Sci Technol. 2026 Mar 25;15(3):25. doi: 10.1167/tvst.15.3.25 (PMC13034998; doi:10.1167/tvst.15.3.25)
Supplement: Supplement 1 [file tvst-15-3-25_s001.pdf]

Supplementary Figure 1 ROC curves comparing the predictive ability of age, demographic models, and RAR for cataract.

RAR: The red cell distribution width to albumin ratio.

The DeLong test indicated that adding RAR to the demographic model significantly improved the predictive performance (AUC 0.8795 vs 0.8783, ***P* = 0.007**).

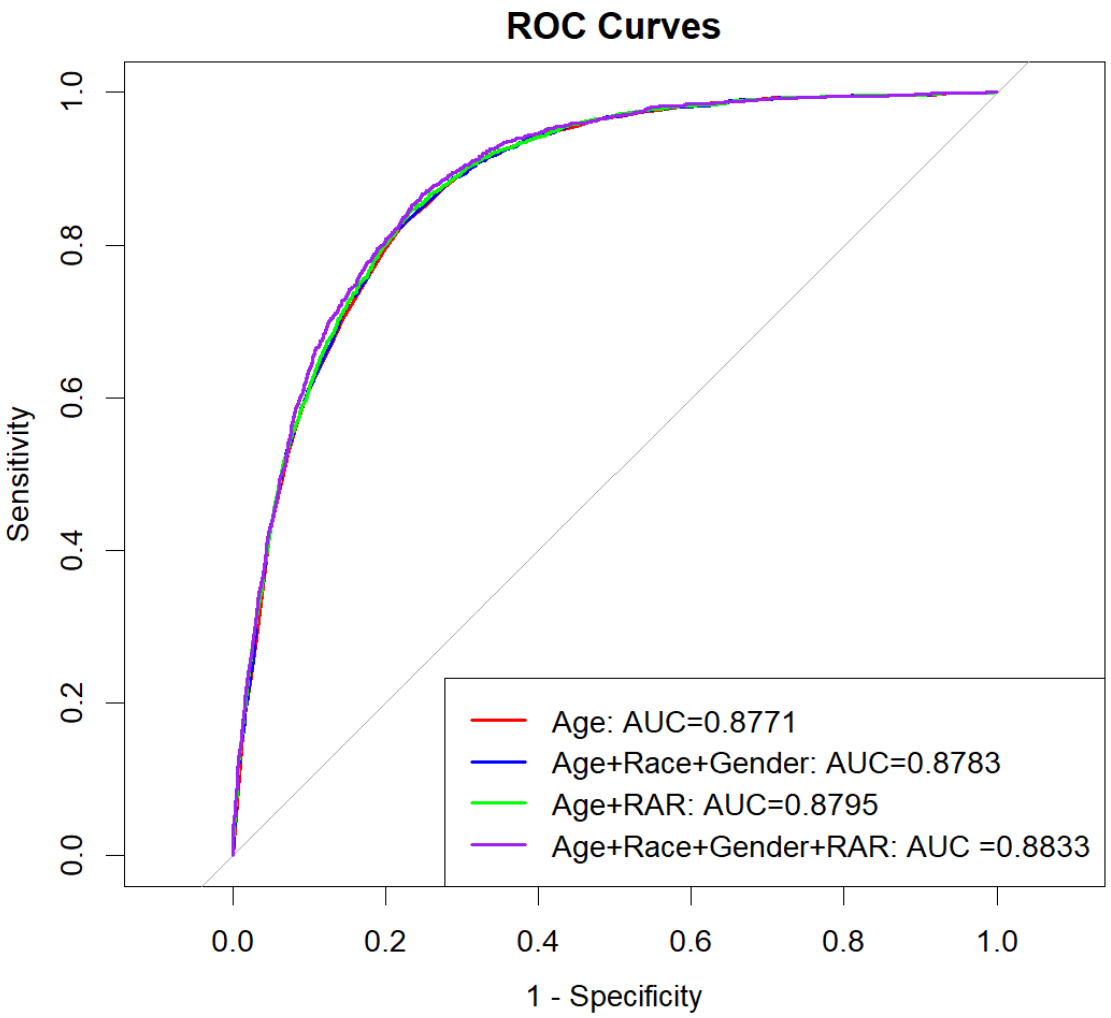

Supplemental Table 1 Sensitivity analyses of the association between RAR and cataract risk.

Item	Age ≥50 years*	Excluding Mediators#
RAR	1.34 (1.13, 1.60), *P* < 0.01	1.38 (1.17, 1.62), *P* < 0.01

Data are presented as OR (95% CI) with *P*-values.

OR, odds ratio; CI, confidence interval; RAR, red blood cell distribution width-to-albumin ratio; Ref, reference category; NA, not applicable.

* Age ≥50 years: Sensitivity analysis restricted to participants aged 50 years or older using the fully adjusted model.

#No Mediators: Sensitivity analysis excluding potential mediators (BMI, hypertension, diabetes, and hyperlipidemia) to assess the robustness of the association against overadjustment.
